# Bifaciality Optimization of TBC Silicon Solar Cells Based on Quokka3 Simulation

**DOI:** 10.3390/ma19020405

**Published:** 2026-01-20

**Authors:** Fen Yang, Zhibin Jiang, Yi Xie, Taihong Xie, Jingquan Zhang, Xia Hao, Guanggen Zeng, Zhengguo Yuan, Lili Wu

**Affiliations:** 1 College of Materials Science and Engineering, Sichuan University, Chengdu 610065, China; 2 Sichuan Sunsync Photovoltaic Technology (Yibin) Co., Ltd., Yibin 644000, China; 3 Institute of New Energy and Low-Carbon Technology, Sichuan University, Chengdu 610065, China

**Keywords:** tunnel oxide-passivated back contact solar cells, bificiality, simulation

## Abstract

**Highlights:**

**What are the main findings?**

- A simulated conversion efficiency of 27.26% and a bifaciality ratio of 92.96% have been achieved for TBC solar cells.

**What are the implications of the main findings?**

- By considering the interaction between parameters, the optimal performance balance point was determined.
- An integrated opto-electrical analysis and design were specifically conducted.

**Abstract:**

Tunnel Oxide-Passivated Back Contact solar cells represent a next-generation photovoltaic technology with significant potential for achieving both high efficiency and low cost. This study addresses the challenge of low bifaciality inherent to the rear-side structure of TBC cells. Using the Quokka3 simulation and assuming high-quality surface passivation and fine-line printing accuracy, a systematic optimization was conducted. The optimization encompassed surface morphology, optical coatings, bulk material parameters (carrier lifetime and resistivity), and rear-side geometry (emitter fraction, metallization pattern and gap width). Through a multi-parameter co-optimization process aimed at enhancing conversion efficiency, a simulated conversion efficiency of 27.26% and a bifaciality ratio of 92.96% were achieved. The simulation analysis quantified the trade-off relationships between FF, bifaciality, and efficiency under different parameter combinations. This enables accurate prediction of final performance outcomes when prioritizing different metrics, thereby providing scientific decision-making support for addressing the core design challenges in the industrialization of TBC cells.

## 1. Introduction

Interdigitated back contact (IBC) and tunnel oxide-passivated contact (TOPCon) solar cells are currently a major research focus in the photovoltaic field. IBC solar cells feature all metallization lines on the rear side, minimizing optical shading losses [1,2,3]. This configuration not only enables high short-circuit current density and efficiency [4,5,6], but also offers superior aesthetics and reliability [7,8]. TOPCon solar cells utilize tunnel oxide-passivated contact technology, which significantly reduces carrier recombination losses and increases the open-circuit voltage, thereby achieving higher conversion efficiency [9,10,11]. Meanwhile, since its conception, the technology has made remarkable progress, and its manufacturing processes have gradually matured [11,12,13]. The TBC solar cell, which integrates IBC and TOPCon manufacturing processes, combines the advantages of both technologies. It achieves high short-circuit current density and efficiency due to the absence of front-side grid shading. The incorporation of a tunnel oxide passivation layer at the rear is compatible with mass production lines, and the associated investment cost is significantly lower than that for heterojunction technology-based back contact (BC) cells, making TBC a crucial research direction for future development [14,15,16,17].

To address the two major bottlenecks of process complexity and low bifaciality, researchers are continuously optimizing TBC cell technology. In studies of other solar cell types, various research groups have adopted differentiated technical pathways to improve bifaciality, achieving remarkable results. For instance, Feng et al. enhanced the bifaciality of a TOPCon cell to 94.3% by employing a rear-side selectively etched pyramid structure combined with a zebra-line passivating contact technology; the corresponding encapsulated module also achieved a bifaciality of 91.7% and a power output of 722.0 W [18]. Xie et al. reported a bifaciality as high as 97% for a heterojunction cell by introducing transparent conductive oxide (TCO)-free and dopant-free electron-selective contact [19]. Elmar Lohmüller et al. achieved a bifaciality of 88% for a PERC cell by optimizing the rear-side silicon nitride coverage [20]. Furthermore, Tong et al. developed a bifacial light management strategy for TBC cells, resulting in a bifaciality of 81.33% [21].

To enhance the bifaciality and conversion efficiency of TBC solar cells, researchers have focused on optimizing rear-side textured structures, metallization patterns, and gap width. For example, Tong et al. proposed a bifacial light management strategy wherein the front surface features a low-damage micro-/sub-micro composite texture, while the rear gap region is designed as a planar surface supplemented with spherical cap-shaped nanostructures [21]. This design significantly improves the light-trapping capability and overall passivation performance of the cell. In another study, Wu et al. achieved a conversion efficiency of 27.3% on a designated area of 243.0 cm^2^ by adopting a 780 µm pitch and a p/n ratio of 1.4, combined with a wafer-edge-coverage technique that offers low recombination current density (J_0_) and low contact resistivity (ρc) [22].

The aforementioned studies have successfully improved bifaciality by optimizing different aspects of the rear-side morphology, passivation quality, and gap region structure in various cell architectures. However, systematic investigations into the full rear-side pyramid morphology, rear metallization layout, and gap width remain relatively limited. Therefore, this work employs the Quokka3 simulation platform to systematically investigate the influence of key parameters—including the front and rear surface morphology, bulk resistivity and carrier lifetime, rear metallization pattern, and gap width—on cell performance. The aim is to achieve co-optimization of the geometric structure and wafer parameters of TBC solar cells. The optimized cell is expected to exhibit significant improvements in both bifaciality and efficiency, thereby providing a theoretical foundation and technical guidance for practical manufacturing.

## 2. Cell Structure and Simulation Methodology

### 2.1. Cell Structure

**Figure 1:**
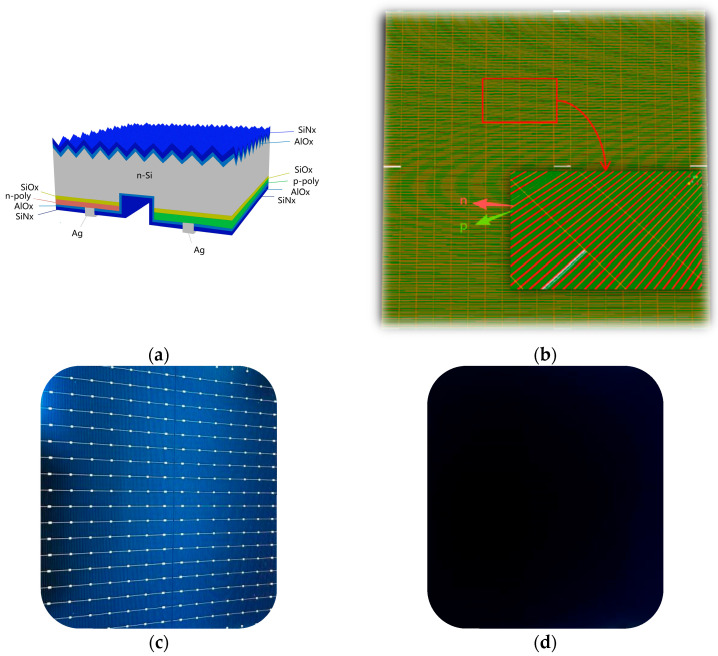
Schematic diagram of the TBC solar cell structure: (**a**) unit structure used for simulation; (**b**) cell structure used for simulation; (**c**) front view of the actual cell; (**d**) rear view of the actual cell.

In this study, the TBC solar cell was simulated with its unit cell and overall structure illustrated in Figure 1. The cell employs an n-type substrate and avoids doping on the front surface. The front-side passivation and anti-reflection layer consists of Al_2_O_3_/SiN_x_, with the thickness optimized via Opal2 simulations. The rear-side structure, from bottom to top, comprises a 1.5 nm SiO_x_ tunneling layer, p-type and n-type poly-Si layers, and a dual-layer stack of Al_2_O_3_/SiN_x_. This rear-side bilayer design provides effective passivation while also enhancing internal photon reflection, thereby improving internal light trapping and overall cell performance.

### 2.2. Simulation Methodology

This study employs Quokka3 software for simulations. While the free version has limited functionality, we utilized a paid academic license, which provides access to all features of the software. The software is specifically designed for rapid simulation of silicon solar cells in one- to three-dimensional spaces. Its core approach utilizes simplified models, such as the quasi-neutral approximation and conducting boundary conditions, which significantly reduce the computational complexity of carrier transport models without substantially compromising generality or accuracy. Consequently, Quokka3 enables the simulation of moderately complex 3D cell geometries on standard computers within a short timeframe, achieving accuracy comparable to advanced commercial simulation tools. The software was used to solve the electrical characteristics of the device and derive typical solar cell performance parameters. A high level of passivation and fine-line printing precision were assumed in the simulations, with specific parameter settings provided in Table 1.

## 3. Results and Discussion

### 3.1. Optical Film Stack Structure

OPAL2 is a specialized optical simulator for photovoltaic solar cell surfaces, capable of precisely calculating surface reflectance, optical layer absorptance, and the resulting spectral distribution of light entering the semiconductor material [23,24]. It can further determine the photogenerated current produced inside the cell under a given incident spectrum. The outputs RAT(λ) and G(z) can be directly used as input parameters for the Quokka3 simulation software. Here, RAT(λ) describes the reflectance, absorptance, and transmittance characteristics at different wavelengths, while G(z) represents the carrier generation rate as a function of depth within the cell. In OPAL2, users can flexibly configure surface morphology (e.g., planar fraction and different types of textured structures), pyramid apex angle, the formula for the Z-parameter in the light trapping model, as well as the number, thickness, material, and deposition process of thin-film layers to obtain the desired results. The software performs internal ray tracing, supports user-defined angles of incidence, and can simulate more complex geometrical configurations.

**Figure 2:**
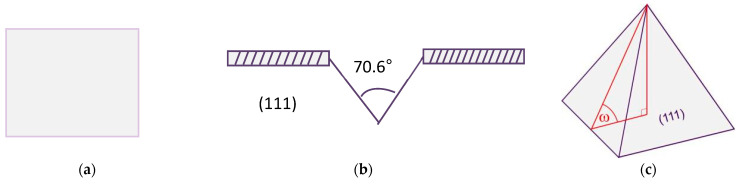

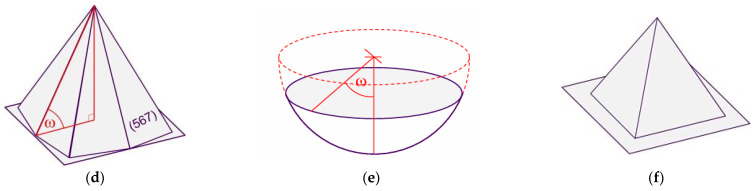
Surface structures and planar fraction distribution of the silicon wafers [23]. (**a**) Planar, (**b**) V grooves, (**c**) Upright pyramids, (**d**) Upright hillocks, (**e**) Spherical caps, and (**f**) Schematic of planar fraction distribution on the textured surface.

Figure 2 shows five surface structures simulated in this study: (a) planar, (b) V-grooves, (c) upright pyramids, (d) upright hillocks, and (e) spherical caps. As illustrated in Figure 2f, the planar fraction may exist independently of the textured regions or be distributed within them. The symbol ω denotes the angle between the pyramid facet and the planar surface.

**Figure 3:**
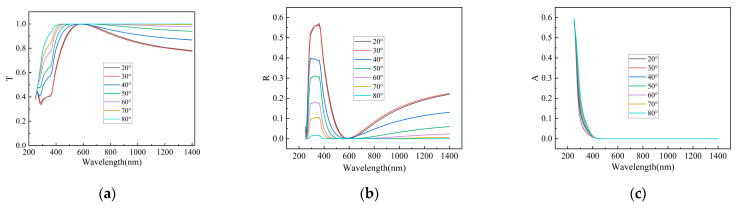

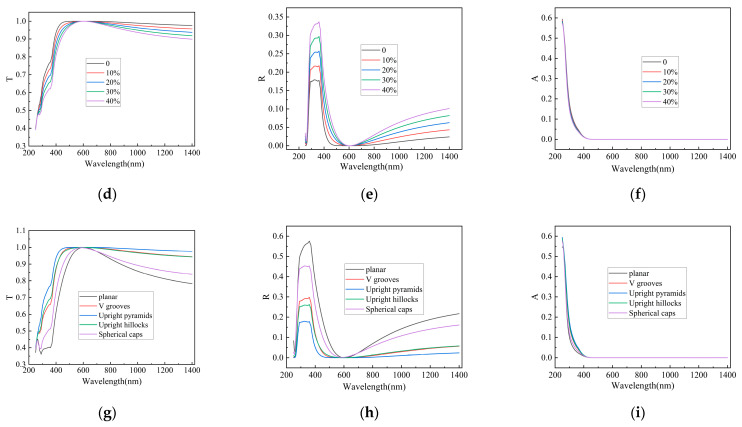
Photon transmission (T), reflection (R), and absorption (A) at the front surface: (**a**) T vs. pyramid apex angle; (**b**) R vs. pyramid apex angle; (**c**) A vs. pyramid apex angle; (**d**) T vs. planar fraction; (**e**) R vs. planar fraction; (**f**) A vs. planar fraction; (**g**) T vs. surface morphology; (**h**) R vs. surface morphology; (**i**) A vs. surface morphology.

Figure 3 illustrates the influence of pyramid apex angle and surface morphology on the photogenerated current of the silicon wafer. As shown in Figure 3a–c, as the angle between the pyramid facet and the base plane increases, photon reflection losses decrease, leading to a greater number of photons entering the bulk region of the cell and thereby enhancing the current output. Considering that the typical apex angle of pyramids formed by anisotropic etching of monocrystalline silicon is 54.74°, and accounting for process variations in actual production, the facet-base angle was set to 60° in simulations to evaluate its potential for optical performance improvement. Figure 3d–f further demonstrate that reducing the planar fraction of the pyramidal texture helps to minimize sunlight reflection and enhance photocurrent. To maximize light trapping, the planar fraction was set to zero in the simulations. Additionally, Figure 3g–i compare the optical behavior of different surface textures. Based on the photocurrent transmitted into the cell, the upright pyramid structure exhibits the best overall performance, making it the most suitable surface morphology for the present cell design.

Furthermore, this study investigated the influence of SiN_x_ layer thickness on cell performance, with the results summarized in Table 2. Simulations indicate that under a fixed SiN_x_ refractive index, variations in thickness primarily alter the reflectance behavior at the front surface, thereby modulating the number of photons entering the cell. The maximum photocurrent entering the bulk material is achieved when the SiN_x_ thickness falls within the range of 65–70 nm. In actual fabrication processes, a relatively thicker SiN_x_ layer (e.g., 70 nm) not only reduces reflection through optical interference effects but also incorporates a higher concentration of hydrogen atoms and provides sufficient fixed positive charges, thereby enhancing passivation quality and significantly suppressing carrier recombination. Consequently, a SiN_x_ thickness of 70 nm was selected as the optimal value in this study.

### 3.2. Different Rear-Side Morphologies

**Figure 4:**
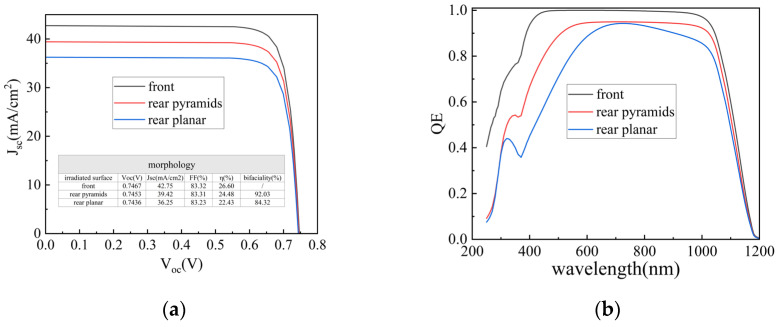
Simulated illumination on TBC solar cells with different rear-side morphologies: (**a**) J-V curve and (**b**) QE curve.

When light is incident from the rear side, severe reflection occurs due to a planar rear structure, resulting in low bifaciality. To address this issue, this study compared the J–V characteristics and quantum efficiency (QE) for planar and pyramidal rear-side morphologies via simulation, and analyzed their impact on bifaciality; the results are shown in Figure 4.

The simulation results reveal significant differences in the J–V and QE curves between planar and pyramidal rear-side textures when the front surface features a pyramidal structure. Since all metallization grids are located on the rear side in TBC cells, light incident from the rear suffers from considerable shading and reflection losses, leading to a notable reduction in short-circuit current density (J_sc_). As observed in Figure 4a, the pyramidal rear texture exhibits lower photon reflection loss compared to the planar structure, thus yielding a higher J_sc_. Furthermore, the results indicate that when the back-side morphology is configured as upright pyramids, the bifaciality factor increases from 84.32% to 92.03%. The QE curves in Figure 4b further indicate a poor short-wavelength response under rear illumination, as most photons in this range are absorbed before reaching the bulk region. A comparison of QE responses under different rear morphologies shows that the pyramidal structure enables more photons in both the 300–700 nm and 700–1000 nm wavelength ranges to enter the cell, thereby improving the collection efficiency of photogenerated carriers.

H. Sharma et al. [25] significantly improved the cell performance by optimizing the surface passivation process, increasing the effective minority carrier lifetime from 2 ms to 4.3 ms, the J_sc_ from 37.8 mA/cm^2^ to 38.5 mA/cm^2^, and the efficiency from 19% to 22.4% [25]. Meanwhile, LONGi introduced dome-shaped nanostructures into the gap regions on the back side of TBC cells, experimentally achieving a bifaciality of only 81.33% [21]. In this study, we further investigated the back-side morphology and achieved a simulated bifaciality of 92.03%. The simulated J–V curves indicate that the modification of the back-side morphology leads to an increase of 3.17 mA/cm^2^ in J_sc_. Comparative analysis with the reported results reveals that such differences indicate there remains significant room for improvement in both J_sc_ and bifaciality under current experimental conditions. The discrepancy between the simulation and the aforementioned experiments stems from differences in surface texture design. In the simulation, upright pyramids were applied across the entire rear surface, which significantly reduces light reflection. In the experiments, however, this pyramid design either requires further optimization or has only been applied for passivation in the rear-side gap regions, resulting in reduced effectiveness in reflection suppression. Therefore, future work should focus on improving the design of the cell surface texture to minimize light reflection and thereby enhance solar cell efficiency.

### 3.3. Bulk Resistivity and Minority-Carrier Lifetime

Carrier lifetime and bulk resistivity are two key parameters determining the performance of silicon solar cells, and they must be co-optimized to achieve high efficiency. Carrier lifetime influences the transport and collection of minority carriers; an excessively short lifetime reduces collection efficiency, while an overly long lifetime increases material and processing costs, thus requiring a balance between performance and cost. Bulk resistivity, which is determined by the doping concentration, involves a trade-off: excessively high doping concentrations can induce Auger recombination, thereby reducing both open-circuit voltage and current, whereas overly low doping increases series resistance and weakens the built-in electric field. Furthermore, impurities introduced during doping may act as recombination centers, thereby affecting the carrier lifetime. This interplay indicates a coupling relationship between the two parameters, necessitating simulation-based determination of their optimal ranges.

**Figure 5:**
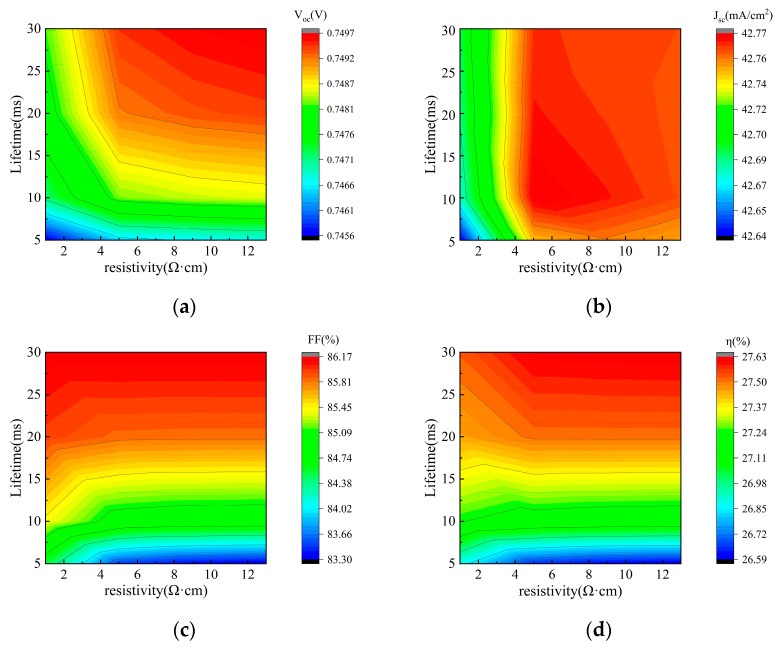
Contour plots of V_oc_, J_sc_, FF, and η simulated by varying bulk resistivity and carrier lifetime under front illumination: (**a**) V_oc_; (**b**) J_sc_; (**c**) FF; (**d**) η.

**Figure 6:**
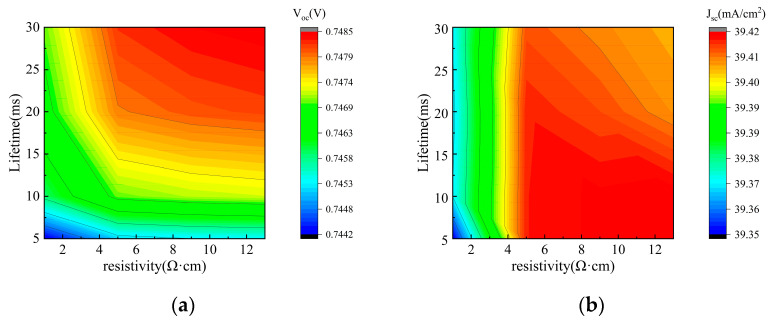

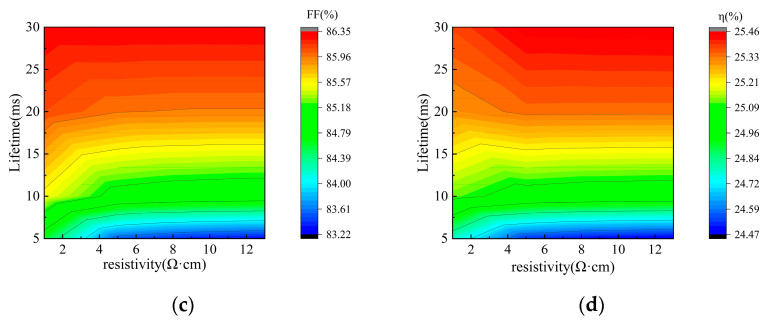
Contour plots of V_oc_, J_sc_, FF, and η simulated by varying bulk resistivity and carrier lifetime under rear illumination: (**a**) V_oc_; (**b**) J_sc_; (**c**) FF; (**d**) η.

In this study, we systematically investigated the influence of carrier lifetime and bulk resistivity on the performance and bifaciality of tunnel oxide-passivated back contact (TBC) solar cells. As shown in Figure 5 (front-side illumination) and Figure 6 (rear-side illumination), the performance variations in the TBC solar cell exhibit the same trend whether under front- or rear-side illumination, and thus both figures are analyzed together. In Figure 5a and Figure 6a, the open-circuit voltage (V_oc_) shows a monotonic rise with longer carrier lifetime, and similarly, the fill factor (FF) in Figure 5c and Figure 6c also increases monotonically. In contrast, J_sc_ in Figure 5b and Figure 6b remains largely stable. Therefore, as shown in Figure 5d and Figure 6d, the overall conversion efficiency (η) is improved.

The relationship between TBC cell performance and bulk resistivity is more complex and is modulated by the carrier lifetime. Specifically, within the low bulk resistivity regime (<5 Ω·cm), the influence of resistivity is strongly dependent on the carrier lifetime. When the lifetime is below 15 ms, increasing the resistivity slightly enhances both V_oc_ and J_sc_; however, this is outweighed by a significant drop in FF, leading to a net decrease in η. In contrast, when the lifetime exceeds 15 ms, increasing resistivity primarily drives a substantial improvement in V_oc_, while FF remains relatively stable, resulting in an overall increase in η. Conversely, when the bulk resistivity exceeds 5 Ω·cm, further increases have a diminishing effect on V_oc_, FF, J_sc_, and η, with all performance parameters showing negligible variation.

These trends can be explained as follows: Carrier lifetime, being an indicator of recombination severity, does not affect the fundamental generation of photogenerated carriers, which explains the insensitivity of J_sc_. In contrast, V_oc_, FF, and η benefit from longer lifetimes. Bulk resistivity is determined by doping concentration. Heavy doping (low resistivity) introduces more impurities, thereby reducing the minority carrier diffusion length. When this length becomes comparable to the cell thickness, carriers are more likely to recombine during transport, causing a slight decrease in J_sc_. Within the low-resistivity regime, the associated heavy doping also reduces the carrier lifetime, creating a coupled relationship that jointly affects cell performance. At high levels of resistance, their influences become relatively independent.

**Figure 7:**
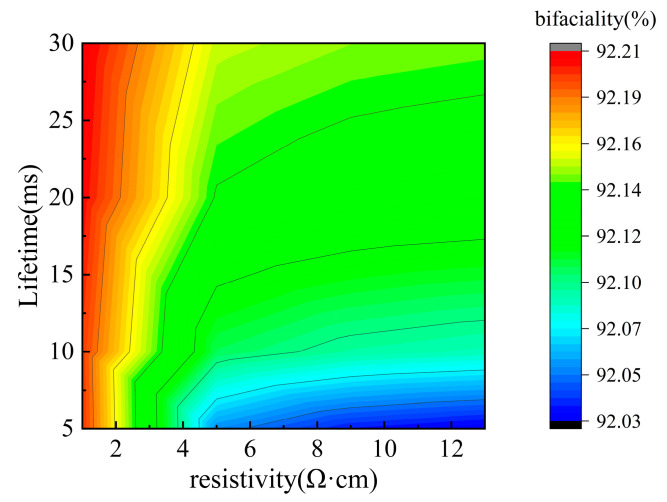
Bifaciality factor of solar cells simulated by varying bulk resistivity and carrier lifetime.

Figure 7 further illustrates the influence of carrier lifetime and bulk resistivity on bifaciality. A higher carrier lifetime leads to increased bifaciality, whereas a higher bulk resistivity results in reduced bifaciality. Considering practical industrial constraints, this study selected a carrier lifetime of 10 ms and a bulk resistivity of 5 Ω·cm as the baseline simulation parameters. To achieve higher bifaciality in mass production, these two parameters must be co-optimized, balancing performance requirements with cost-effectiveness.

S. Bhattacharya et al. demonstrated significant fill factors and efficiency gains in silicon heterojunction solar cells using lower-resistivity n-type wafers [26]. But C. Rittmann et al. found that the effective carrier lifetime increases with rising wafer resistivity, thereby significantly improving solar cell efficiency [27]. The aforementioned study indicates that bulk resistivity and carrier concentration exhibit distinct trends in their influence on solar cell performance. A lower bulk resistivity enhances cell performance, whereas higher resistivity favors longer carrier lifetimes, thereby improving cell efficiency. Thus, determining how bulk resistivity and carrier lifetime affect solar cell performance is particularly important. Based on the simulation results shown in Figure 5d and Figure 6d, when the carrier lifetime ranges from 5 to 10 ms, cell efficiency decreases as bulk resistivity increases. When the carrier lifetime falls within the range of 10 to 20 ms, the impact of bulk resistance on efficiency is negligible. However, when the carrier lifetime reaches the range of 20 to 30 ms, efficiency significantly increases with higher bulk resistance. These results demonstrate that the effect of resistivity strongly depends on the range of carrier lifetimes. Therefore, apparent contradictions in experimental findings can be explained by differences in the carrier lifetime range, as the influence of bulk resistivity on solar cell efficiency varies accordingly.

### 3.4. Gap Region Width

**Figure 8:**
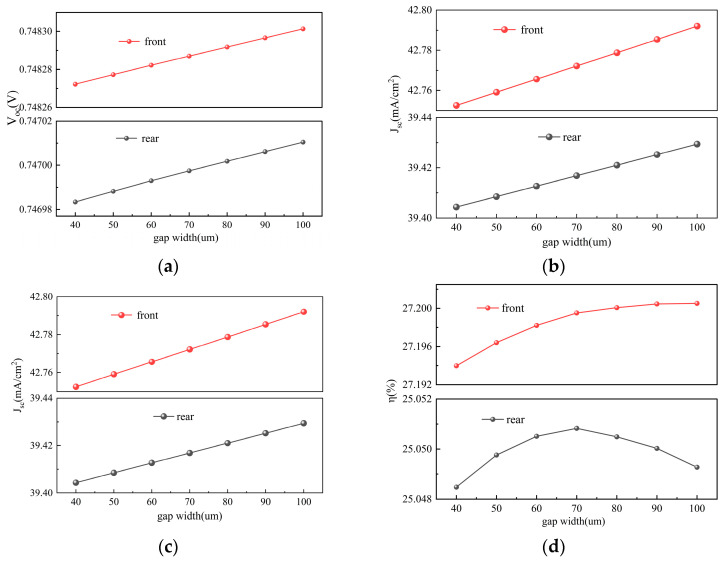
Contour plots of V_oc_, J_sc_, FF, and η simulated by varying the gap region width under front and rear illumination, respectively: (**a**) V_oc_; (**b**) J_sc_; (**c**) FF; (**d**) η.

**Figure 9:**
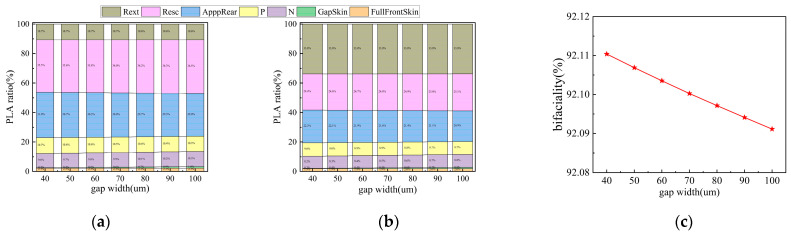
PLA loss analysis and bifaciality factor were simulated by varying the gap region width under front and rear illumination, respectively: (**a**) front illumination; (**b**) rear illumination; (**c**) bifaciality.

The gap width is a key geometric parameter influencing the rear-side light-receiving performance of TBC solar cells. To investigate its impact, this study simulated the effects of varying the gap width within the range of 40–100 μm on cell performance and bifaciality. As shown in Figure 8a,b, both V_oc_ and J_sc_ exhibit an increasing trend with the widening of the gap width. Meanwhile, according to Figure 8c, FF shows a decreasing trend as the gap width increases. Consequently, as indicated in Figure 8d, η demonstrates a trend of initially increasing and then decreasing. As shown in Figure 8d, under front-side illumination, the conversion efficiency (η) reaches its maximum at a gap width of 90 μm, whereas under rear-side illumination, the peak efficiency occurs at 70 μm. The optimal gap width for maximum efficiency varies under illumination from different sides. Therefore, it is necessary to discuss the bifaciality under different gap widths. As indicated in Figure 9c, the bifaciality of the cell gradually decreases with increasing gap width.

The observed trends can be explained as follows: Increasing the gap width helps reduce the risk of inter-electrode short circuits and tunneling recombination, thereby decreasing the dark current and improving V_oc_. Simultaneously, a wider gap expands the high-quality passivation area and reduces the “dead area” caused by metal coverage, thus enhancing carrier collection efficiency and increasing J_sc_. However, a larger gap width also elongates the lateral transport path for carriers, leading to increased series resistance and a consequent decrease in FF. Since η is the product of J_sc_, V_oc_, and FF, its variation is determined by the combined effect of these three parameters. When the gap width is relatively small, appropriately increasing it significantly reduces leakage and recombination losses, such that the gains in J_sc_ and V_oc_ far outweigh the slight decline in FF caused by increased resistance, resulting in an overall improvement in efficiency. However, when the gap width is further increased, the continued rise in series resistance causes the decline in FF to gradually exceed the gains from J_sc_ and V_oc_, leading to a subsequent drop in efficiency. Figure 9a,b further reveal the sources of power loss under different gap widths. A comparison of the two figures indicates that optical reflection is the primary contributor to power loss, with reflection from the metal grids being the most significant factor, while the impacts of escape reflection and parasitic absorption are relatively minor. Therefore, setting the gap width requires careful consideration of whether bifaciality or front-side efficiency is the primary objective. After comprehensively evaluating both factors, a gap width of 75 μm was selected for subsequent simulations. At this point, the efficiency growth rate had already begun to slow, and the bifaciality remained close to 92.1%.

### 3.5. Emitter Ratio and Finger Pitch

**Figure 10:**
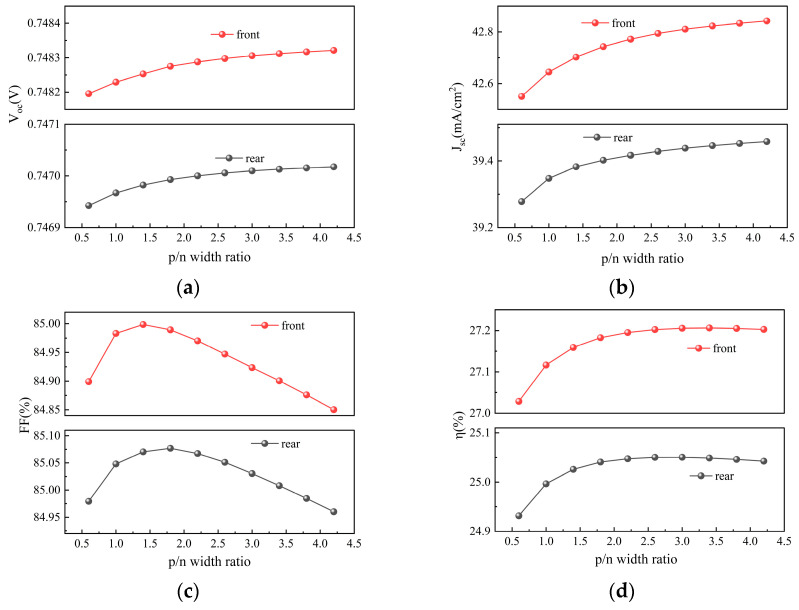
Contour plots of V_oc_, J_sc_, FF, and η simulated by varying the p/n width ratio under front and rear illumination, respectively: (**a**) V_oc_; (**b**) J_sc_; (**c**) FF; (**d**) η.

**Figure 11:**
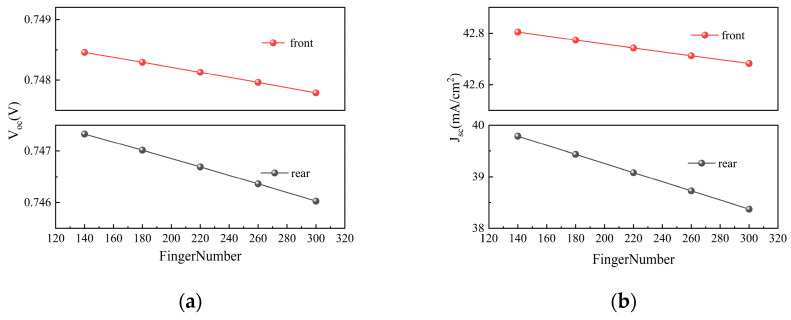

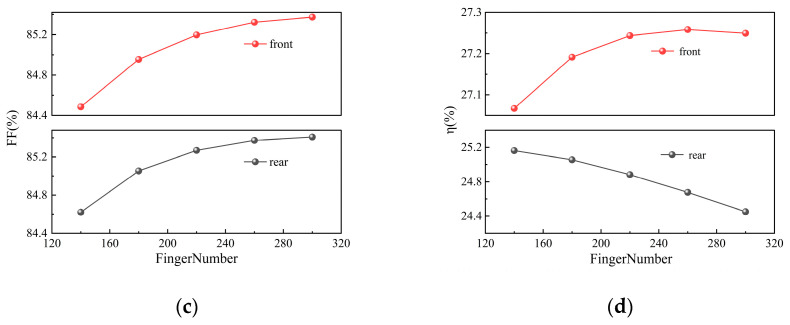
Contour plots of V_oc_, J_sc_, FF, and η simulated by varying the finger number under front and rear illumination, respectively: (**a**) V_oc_; (**b**) J_sc_; (**c**) FF; (**d**) η.

**Figure 12:**
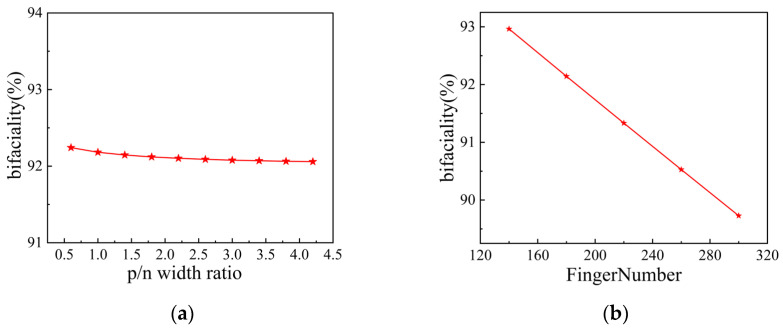
Bifaciality factor simulated by varying the p/n width ratio and the finger number: (**a**) the p/n width ratio; (**b**) the finger number.

The emitter fraction and total pitch are key geometric parameters affecting the performance of TBC silicon solar cells. The emitter fraction is determined by the width ratio of the p-type to n-type regions, while the total pitch depends on the number of rear-side grid lines. This study systematically investigates the effects of the p/n width ratio (ranging from 0.6 to 4.2) and the number of grid lines (ranging from 140 to 300) on cell performance and bifaciality. Figure 10 shows the influence of the p/n width ratio on solar cell performance, Figure 11 illustrates the impact of the number of grid lines on solar cell performance, and Figure 12a,b present the corresponding bifaciality variations with changes in the p/n width ratio and the number of grid lines, respectively.

As shown in Figure 10a,b, under both front- and rear-side illumination, V_oc_ and J_sc_ show an increasing trend as the p/n width ratio rises, though their growth rates gradually slow down. From Figure 10c, it can be seen that as the p/n width ratio increases, FF first rises and then declines, reaching its peak at a ratio of approximately 1.5. Consequently, η in Figure 10d exhibits an initial increase followed by a slight decrease. Figure 12a reflects the variation in bifaciality with the p/n ratio: as the ratio increases, bifaciality declines rapidly before eventually stabilizing.

These performance variations originate from the competing mechanisms of carrier collection and series resistance. At low p/n ratios, the wider n-region ensures efficient electron collection, but the overly narrow p-region restricts hole transport and intensifies recombination. As the p/n ratio increases, the broadening p-region effectively enhances hole collection efficiency, leading to an increase in J_sc_, while reduced recombination contributes to higher V_oc_. However, once the hole collection approaches saturation, the performance gains gradually diminish. Regarding FF, as the p/n ratio increases from a low value, the average lateral transport distance for holes shortens, reducing transport resistance and optimizing series resistance near a ratio of 1.5. Further increasing the p/n ratio, however, narrows the n-region excessively, resulting in insufficient metal–semiconductor contact area, increased electron contact resistance, higher overall series resistance, and consequently, a decline in FF. Ultimately, the trend in η is initially dominated by improvements in V_oc_ and J_sc_, then gradually overtaken by the subsequent decline in FF, resulting in an initial increase followed by a slight decrease.

As shown in Figure 11a,b, both V_oc_ and J_sc_ gradually decrease as the number of grid lines increases. Figure 11c indicates that FF continuously rises with an increasing number of grid lines. Figure 11d indicates that under front-side illumination, η shows a trend of first increasing and then decreasing, reaching its maximum of 27.26% when the number of grid lines is 260. In contrast, under rear-side illumination, the efficiency declines continuously, with a noticeably faster rate of decrease compared to front-side illumination. Figure 12b further demonstrates that the bifaciality of the cell decreases rapidly as the number of grid lines increases, and when the number of grid lines continues to decrease, the bifaciality can reach 92.96%.

The variation in cell performance with the number of grid lines stems from a trade-off among carrier transport, optical shading, and electrical recombination. Although increasing the number of grid lines shortens the lateral transport distance for carriers, reducing series resistance and thereby improving FF, it also enlarges the rear metal shading area, reducing photogenerated carrier generation and leading to a decrease in J_sc_.

Simultaneously, the increased metal–semiconductor contact area intensifies interface recombination, raising the dark saturation current and consequently reducing V_oc_. Under front-side illumination, when the number of grid lines is low, series resistance dominates the performance limitation. At this stage, the FF gain from adding grid lines outweighs the losses in V_oc_ and J_sc_, resulting in an increase in efficiency. As grid lines are further densified, the influence of series resistance diminishes, while the V_oc_ and J_sc_ losses caused by recombination and shading become dominant, leading to a subsequent decline in efficiency. Under rear-side illumination, incident photons must first pass through the rear grid structure, making the shading effect more pronounced. Since J_sc_ is already relatively low, the improvement in FF from adding grid lines is limited and cannot compensate for the substantial loss in J_sc_, thus causing efficiency to decline continuously. In summary, the differing responses of front- and rear-side efficiencies lead to a monotonic decrease in cell bifaciality as the number of grid lines increases.

Currently, extensive research has also been conducted in the field of silver paste printing technology, primarily focusing on reducing silver usage. For example, Zhang Yu et al. investigated a low-silver metallization design scheme aimed at significantly reducing silver paste consumption in TOPCon while maintaining high efficiency [28]. M. Krejci et al. primarily studied the microscopic interactions between silver–aluminum paste and the silicon emitter in TOPCon, as well as their impact on cell performance [29]. However, experiments related to the number of grid lines have rarely been reported. Therefore, in this study, we conducted in-depth research and analysis on the number of grid lines in TBC solar cells. Our simulation results indicate that a higher number of grid lines facilitates better carrier collection, thereby enhancing solar cell performance. Thus, it is necessary to strike an appropriate balance in silver paste usage while ensuring both efficiency and cost-effectiveness. Our simulation results provide a balanced reference to determine the optimal range for the number of grid lines in solar cell production. In subsequent production design, targeted adjustments can be made based on the variation patterns and influencing mechanisms of the above parameters, thereby maintaining high efficiency while achieving relatively high bifaciality.

Based on the simulation of the aforementioned parameters, maximum efficiency and bifaciality are achieved under different parameter configurations. Therefore, Table 3 summarizes all parameters under various conditions, aiming to provide directions for subsequent experiments and highlight key priorities for other researchers.

## 4. Conclusions

This study centered on the core objective of improving the conversion efficiency and bifaciality of TBC solar cells, carrying out a series of systematic simulations and mechanism investigations. The main conclusions are as follows: Adopting an upright pyramid texture on both the front and rear surfaces increased the simulated bifaciality to 92.03%. Taking into account both performance and manufacturability, a SiN_x_ thickness of 70 nm, a carrier lifetime of 10 ms, and a bulk resistivity of 5 Ω·cm were identified as the optimal parameter combination for achieving the best balance of performance. To pursue both high efficiency and high bifaciality, the gap width was ultimately selected as 75 μm. To achieve the highest fill factor or the highest front-side conversion efficiency, the p/n width ratio should be set to 1.5 or 3.4, respectively. Considering the impact of both factors, the final chosen p/n width ratio was 2.6. Furthermore, under illumination, the front-side efficiency reached a peak of 27.26% at 260 grid lines, corresponding to a bifaciality of 90.7%. When the number of grid lines was reduced to 140, bifaciality reached 93%.

In summary, through multi-parameter and multi-dimensional simulation and optimization, this study significantly improved the overall performance of TBC cells and clarified the interaction mechanisms between various parameters. It provides a clear technical pathway and theoretical foundation for resolving the typical trade-off between “high efficiency” and “high bifaciality,” offering valuable insights for guiding subsequent process development and industrial mass production.

## Figures and Tables

**Table 1:** Simulation parameters configured in Quokka3.

Parameter	Value	Parameter	Value
CellThickness	130 μm	Bulk Resistivity	1–13 Ω·cm
Rseries	0.001 Ω·cm^2^	Lifetime	5–30 ms
Rshunt	10^6^ Ω·cm^2^	FSF Non-Contact J_0_	0.3 fA·cm^−2^
P-poly Thickness	340 nm	Emitter Contact J_0_	10 fA·cm^−2^
P-poly DopingDensity	5 × 10^19^ cm^−3^	Emitter Non-Contact J_0_	0.3 fA·cm^−2^
N-poly Thickness	280 nm	BSF Contact J_0_	1 fA·cm^−2^
N-poly DopingDensity	3 × 10^20^ cm^−3^	BSF Non-Contact J_0_	0.3 fA·cm^−2^
Al_2_O_3_ Thickness	5 nm	Emitter Width	693–322 μm
SiN_x_ Thickness	70 nm	BSF Width	165–537 μm
NFinger Width	26 μm	FingerNumber	140–300
NFinger Width	27 μm	BusbarNumber	10
Busbar Width	114 μm	GapSkin Width	40–100 μm

**Table 2:** Effect of SiN_x_ Thickness on Front Surface Reflectance, Thin-Film Absorptance, and Photocurrent Density in the Substrate.

SiNx Thickness (nm)	J_R_ (mA/cm^2^)	J_A_ (mA/cm^2^)	J_T_ (mA/cm^2^)
55	0.46	0.07	43.22
60	0.41	0.07	43.27
65	0.39	0.07	43.30
70	0.39	0.07	43.29
75	0.41	0.07	43.27
80	0.46	0.08	43.22

**Table 3:** Key Parameters for achieving high efficiency and high bifaciality, respectively.

Rear-Side Morphologies	SiN_x_ Thickness (nm)	Bulk Resistivity (Ω·cm)	Minority-Carrier Lifetime (ms)	Gap Region Width (μm)	p/n Width Ratio	Figure Number	η (%)	Bifaciality (%)
Upright pyramids	70	5	10	75	2.6	260	27.26	90.7
Upright pyramids	70	5	10	75	2.6	140	27.06	93

## Data Availability

The original contributions presented in this study are included in the article. Further inquiries can be directed to the corresponding authors.
